# Reply to ‘Comment on “Tissue-infiltrating immune cells as prognostic markers in oral squamous cell carcinoma: a systematic review and meta-analysis”’

**DOI:** 10.1038/s41416-019-0460-3

**Published:** 2019-04-23

**Authors:** Elin Hadler-Olsen, Anna Maria Wirsing

**Affiliations:** 1 Department of Medical Biology, Faculty of Health Sciences, University of Tromsø – The Arctic University of Norway, 9037 Tromsø, Norway; 2 Department of Clinical Dentistry, Faculty of Health Sciences, University of Tromsø – The Arctic University of Norway, 9037 Tromsø, Norway; 3 Department of Clinical Pathology, University Hospital of North Norway, 9038 Tromsø, Norway

**Keywords:** Head and neck cancer, Prognostic markers

We would like to thank you for the opportunity to respond to the issues raised in the letter by Dr Zhou and colleagues. We are also grateful to Dr Zhou and colleagues for their interest in our work.

In their letter to the editor, Dr Zhou and colleagues raise concerns about the choice of databases in our literature search. We limited our search to Medline and Embase, and additionally searched the Cochrane Library and reference lists of reviews on related topics. Indeed, a well-conducted systematic review requires a broad coverage of the literature to identify as many relevant studies as possible. According to the Cochrane Handbook, The Cochrane Central Register of Controlled Trials (CENTRAL), Medline and Embase are considered the most important bibliographic sources for inclusion in systematic reviews on intervention studies or studies on the accuracy of diagnostic tests.^1^ Prognostic marker studies are usually explorative, retrospective studies and not clinical intervention studies,^2^ which is why we think that searching The Cochrane CENTRAL and ClinicalTrials.gov, as suggested by Zhou and colleagues, is likely to be of limited benefit. Searching Web of Science in addition to Medline and Embase in combination with Google Scholar, was recommended in a recent study by Bramer et al.,^3^ but has to the best of our knowledge not been found earlier. To date, no consensus exists on the type and number of electronic databases that should be searched, and it has been shown that the vast majority of relevant articles can be retrieved from a limited number of databases.^4,5^ Finally yet importantly, review authors have to balance the extensiveness of the search with the efficiency in use of time and budget.^1^

Dr Zhou and colleagues also raise concerns about the assessment of publication quality and risk of bias. We agree with Dr Zhou and colleagues that evaluating risk of bias is important to estimate the effect of the results. However, as outlined in the PRISMA Statement, this can be assessed by many methods, such as scales, checklists, and individual components.^6^ We utilized a checklist adapted from the REMARK guidelines^7^ to evaluate the reporting quality of the studies. These guidelines were designed to promote proper reporting in prognostic marker research and not as a tool to assess risk of bias. Therefore, we evaluated both the reporting quality and the risk of bias in a qualitative fashion without providing a quantitative grading score for the individual articles.

Dr Zhou and colleagues point out that our results should be interpreted with caution, and we have indeed stated that the conclusions derived from our review are uncertain.^8^ This is mostly because of reporting deficiencies on study design and conduct in the included studies, which inflict substantial risk of selection, performance and detection bias. Although CD163+ M2 macrophages and CD57+ natural killer cells were the most promising predictors of survival in oral cancer patients, we have concluded that these results should be validated in large, standardized studies before they are implemented in the clinic. We thank Dr Zhou and colleagues for valuable comments, but we do not believe that using a different methodological approach with regard to both literature search and assessment of risk of bias would change this conclusion. We hope that focusing towards proper and transparent reporting of biomarker studies will increase their quality and clinical usefulness. Summarizing the current knowledge, although it is hampered by uncertainty, may help direct future research.
